# Make the Unconscious Explicit to Boost the Science of Consciousness

**DOI:** 10.3389/fpsyg.2020.00260

**Published:** 2020-02-19

**Authors:** Johan Eriksson, Aurelie Fontan, Tiziana Pedale

**Affiliations:** ^1^Umeå Center for Functional Brain Imaging (UFBI), Umeå University, Umeå, Sweden; ^2^Physiology Section, Department of Integrative Medical Biology, Umeå University, Umeå, Sweden

**Keywords:** consciousness, unconscious, false negative, neural correlates of consciousness, high-level cognition

## Introduction

If we are to understand the how's and why's of conscious processing we need to compare it with unconscious processing and see how they differ (Baars, 1988). An accurate characterization of unconscious processing is therefore crucial for continued progression in the science of consciousness. The scope and limits of unconscious processing have been extensively investigated for more than a century (see e.g., Kouider and Dehaene, 2007, for a historical perspective), but a consensus has yet to emerge. While some argue that unconscious processing is very limited (e.g., Holender and Duscherer, 2004), others have proposed that anything that can be done consciously can also be done unconsciously (Hassin, 2013; but see Hesselmann and Moors, 2015). The controversy continues in part for methodological/technical reasons, for example that it is non-trivial to present a stimulus below the limen of consciousness while maintaining the possibility for the brain to process the information. We argue here that this aspect is particularly relevant to consider when evaluating the possibility of unconscious high-level cognitive functions: if a certain level of processing of the input stimulus cannot be verified to start with, a lack of related high-level cognition would be expected. Such situation provides little evidence against the possibility of a particular type of unconscious high-level cognition. We also argue that evidence against particular unconscious high-level functions should be framed in an experimental context where at least the conscious analog can be demonstrated—otherwise the evidence is again weak. Lastly, we note that an explicit characterization of the unconscious is crucial also in research on the neural correlates of consciousness.

## Appropriate Benchmarks Increase the Weight of Evidence Against High-Level Unconscious Cognition

According to several studies, unconscious processing can occur at high perceptual (see Kouider and Dehaene, 2007) and cognitive levels (see Soto and Silvanto, 2014). However, other studies have failed to provide support for high-level unconscious processing (e.g., Schlossmacher et al., 2017; Biderman and Mudrik, 2018). Demonstrating unconscious processing at *any* level is non-trivial, in the sense that it cannot be taken for granted that a stimulus has been registered and processed simply because it has been “unconsciously” presented to the participant; it may be that the signal is too weak to be processed at all. Different techniques to render stimuli unconscious seem to allow processing at different levels (Breitmeyer, 2015). For example, Fahrenfort et al. (2017) found that the attentional blink allowed perceptual integration of unseen Kanizsa inducers, whereas backward masking did not.

There are many possible reasons for null findings (e.g., lack of power, methodological confounds, inappropriate choice of paradigm) but in the context of unconscious high-level cognition, attempts should be made to at least demonstrate unconscious processes at some relevant level to substantiate claims that a particular high-level function cannot be implemented unconsciously. For example, Schlossmacher et al. (2017) investigated whether facial expressions of emotions could be processed unconsciously during continuous flash suppression. Despite high power, individual adjustment of stimulus contrast, and concurrent measurement of brain activity (which may pick up processes not evident in behavior), they found no difference between neutral and positive/negative facial expressions. Crucially, they did not include a low-level control condition (e.g., scrambled faces, or other appropriate catch trials, see Moors et al., 2019), and it is thus unclear if the null effects were due to an inability to process facial expressions as such, or if the experimental setup did not allow for lower-level processing either, an issue which they themselves discuss extensively (see also Sterzer et al., 2014). They do show some signal change during unconscious trials (Figure 4 in Schlossmacher et al., 2017), but without an appropriate reference condition it is unclear if this change is due to unconscious processing, anticipation, buildup from the Mondrian patterns used during the continuous flash suppression procedure, or something else.

Similarly, Biderman and Mudrik (2018) assessed unconscious object-scene integration using a masking procedure combined with priming in an attempt to replicate previous findings (Mudrik et al., 2011; Mudrik and Koch, 2013). They evaluated whether an unconscious prime including either a congruent or an incongruent object, could influence behavioral performances to categorize a target scene as congruent or incongruent. They did not observe any priming effect and thus claimed no evidence for unconscious integration of objects and scenes. However, as for Schlossmacher et al., Biderman and Mudrik did not include a reference condition such that lower-level processing could be verified. They did include a “visibility post-test” where the same stimulus set was presented either upright or inverted. Here, participants could perform better than chance, which the authors interpreted as indicating partial awareness, in accordance with objective criteria for unconscious perception. Alternatively, one could argue that the upright/inverted task requires a lower level of processing compared to the object-scene integration task and better-than-chance performance could, combined with the subjective measure used in parallel by Biderman and Mudrik, also be interpreted as indicating lower-level unconscious processing. Inclusion of a dedicated reference condition to specifically probe low-level unconscious processing (e.g., a condition where target and prime are identical, to benchmark priming) within the same experiment could have provided valuable information in this regard.

In an earlier attempt to replicate unconscious object-scene integration Moors et al. (2016) also did not find support for integration, but demonstrated a correlation between the time of breaking suppression and low-level image properties. Had there been evidence also of integration, the correlation with low-level image properties could have turned out to be problematic because of the risk of confounding integration with non-relevant image properties (Moors et al., 2019). However, in the context of null support for integration, the correlation with specific image properties could be considered evidence that at least the image content was processed unconsciously. Are the findings by Moors et al. (2016) thereby sufficient to claim that object-scene integration cannot be performed unconsciously? Ideally, for the specific question of object-scene *integration*, one would like to have seen evidence not only of any form of unconscious image processing, but for object identification and scene identification specifically—only then would it be possible to verify/refute integration of the two. This is a tall order indeed, but this issue illustrates that what constitute an “appropriate benchmark” or “relevant processing level” may differ depending on the research question.

A further point to consider when investigating higher-order processing of unconscious information is if the experimental design can elicit the conscious analog of the process of interest. For example, in a recent magnetoencephalography (MEG) working-memory study, Trübutschek et al. (2019) used a masking procedure to present a barely visible square in different locations, with 20% of the trials being “target absent” trials (which constitutes a reference condition to probe also low-level unconscious processing, as discussed above). The task consisted of maintaining the location of the target square and, after an instruction cue, indicate the original position or mentally rotate the remembered position clockwise or anticlockwise. Thus, on rotation trials the participants were required to manipulate the content held in short-term memory, thereby making it possible to test whether a key feature of working memory (i.e., manipulation) also can be performed unconsciously. The behavioral results revealed better-than-chance performance on unconscious trials, supporting the possibility to maintain and manipulate unconscious information. Neural markers of conscious processing were revealed during the maintenance of conscious compared with unconscious and absent trials, but no difference was evident between unconscious and absent trials, suggesting “activity silent” memory storage (Trübutschek et al., 2017; but see Bergström and Eriksson, 2014, 2018; King et al., 2016). Crucially, neural activity after the presentation of the instruction cue (i.e., during manipulation) revealed no difference between conscious or unconscious vs. absent trials. These results were interpreted as efficient short-term unconscious storage, but a conscious reinstatement of the remembered position to allow the subsequent manipulation of the (until then) unconscious information.

However, the fact that there was no MEG-signal difference between the conscious and absent conditions gives room for an alternative interpretation, namely that the experimental paradigm was suboptimal for characterizing manipulation-related activity. Specifically, it seems that the absent trials required a similar load on relevant cognitive processing as conscious (and unconscious) trials, making the neural markers of manipulation indistinguishable between conditions. Since there was no marker for conscious manipulation of working-memory content, it is not surprising that there was none for unconscious manipulation either. Trübutschek et al. may of course be right in their interpretation, but since there was no explicit demonstration of manipulation-related brain activity at all, the conclusion that unconscious memory representations need to be recast as conscious representations to be manipulable remains speculative.

## Neural Correlates of Conscious and Unconscious Processing

An explicit characterization of unconscious processes is also highly desirable in research on the neural correlates of consciousness (NCC). A consensus has been building over the last decade, stating that many traditional paradigms risk conflating the actual NCC with pre-requisite (e.g., attention) and consequent (e.g., reporting) processes (e.g., Aru et al., 2012; Koch et al., 2016). Accordingly, efforts have been made to reduce influence from such confounds, for example through the development of no-report paradigms (see Tsuchiya et al., 2015). However, it remains common practice to assume unconscious processing rather than to explicitly demonstrate it. Unless the neural correlates of *un*conscious processes (NCU) are explicitly characterized and compared with conscious processes, any statement of a NCC will remain hypothetical. Unfortunately, the NCU is many times left uncharted.

For example, when using binocular rivalry (or other bistable stimuli) the experimental rationale is usually that neural activity that follows the shifts in conscious perception rather than the timing of the stimulus itself (which is constant) will mark a NCC (an assumption previously made also by one of the authors). However, not all neural processes that correlate with perceptual switching need to be related to consciousness, as noted previously (Aru et al., 2012; De Graaf et al., 2012; Blake et al., 2014; Giles et al., 2016). Indeed, it was recently demonstrated that binocular rivalry can occur also for unconscious stimuli (Zou et al., 2016). Although there are examples where activity related to the suppressed stimulus is also characterized (e.g., Fries et al., 1997; Tononi et al., 1998), there are other techniques to dissociate stimulus parameters and conscious perception than bistable stimuli that may be better suited to investigate the NCC, for example by using threshold stimuli, manipulations of attention, blindsight, etc. (see e.g., Kim and Blake, 2005; Giles et al., 2016).

A similar argument can be made for the nowadays relatively common approach of manipulating the state of consciousness—usually referring to the level of wakefulness—to investigate the NCC (see Macdonald et al., 2015). The manipulation of the state of consciousness has brought new insight into the NCC by comparing the cognitive and/or brain changes associated with different levels of wakefulness (e.g., sedation vs. no sedation; Boveroux et al., 2010), or during conscious-to-unconscious state transitions (e.g., Cavanna et al., 2018). However, one crucial factor to take into account for the investigation of the NCC is that the manipulation of the global state of consciousness will not only change conscious processes but might also alter several other brain functions. Thus, one cannot rule out that a reduced level of wakefulness could also affect unconscious processing. Until the relationship and/or the differences between conscious and unconscious states are better understood, it seems prudent to control for effects that are not specific to conscious processing (Koch et al., 2016). To be able to assert a neural correlate as related specifically to conscious processing, one could manipulate both content (awareness) and state of consciousness (wakefulness) within the same experiment (Overgaard and Overgaard, 2010; Bachmann and Hudetz, 2014).

## Concluding Remarks

Progress in any field of science hinges on reproducible findings, no less so for a science of consciousness. Arguably, throughout the history of research on unconscious processes there has been more focus on possible false positive findings compared to false negatives. However, true progress requires a handle on both. Here we have argued that extra considerations should be taken in relation to research on unconscious higher-level cognition, such that the weight of evidence from a null finding will increase greatly if presented in a context of appropriate benchmarks. This includes verification that the experimental paradigm can and do support basic processes required for the relevant higher-level process. For example, when studying high-level integration or high-level perceptual processing, a relevant benchmark would be to first show that the information to be integrated/processed is at least discriminated by the brain. Due to the non-trivial nature of implementing an unconscious stimulus presentation, such basic-level processing should not be taken for granted and appropriate verification should be included in each experiment.

By explicitly demonstrating basic-level unconscious processes, the risk of false negative findings can be reduced. We have also argued that there is much to gain by explicitly demonstrating neural correlates of unconscious processes, which can reduce the risk of false positive NCCs. Much has previously been written on the issue of making conscious and unconscious conditions comparable. It remains unclear if this is at all possible, but we here advocate that *at the very least*, neural correlates of unconscious processes should be explicitly demonstrated to, through comparison, substantiate a NCC. Unfortunately, this is not common practice when using bistable stimuli, or when manipulating the state of consciousness.

In conclusion, we believe that progress will increase in the science of consciousness if unconscious processes are made explicit, in the sense that relevant data (behavioral or neural) are explicitly reported to support what are currently all too often implicit background assumptions.

## Author Contributions

JE, AF, and TP wrote the manuscript.

### Conflict of Interest

The authors declare that the research was conducted in the absence of any commercial or financial relationships that could be construed as a potential conflict of interest.
